# Family Environment, Neurodevelopmental Risk, and the Environmental Influences on Child Health Outcomes (ECHO) Initiative: Looking Back and Moving Forward

**DOI:** 10.3389/fpsyt.2020.00547

**Published:** 2020-06-19

**Authors:** Nicole R. Bush, Lauren S. Wakschlag, Kaja Z. LeWinn, Irva Hertz-Picciotto, Sara S. Nozadi, Sarah Pieper, Johnnye Lewis, Dominik Biezonski, Clancy Blair, Julianna Deardorff, Jenae M. Neiderhiser, Leslie D. Leve, Amy J. Elliott, Cristiane S. Duarte, Claudia Lugo-Candelas, T. Michael O’Shea, Lyndsay A. Avalos, Grier P. Page, Jonathan Posner

**Affiliations:** ^1^Department of Psychiatry, Weill Institute for Neurosciences, University of California, San Francisco, CA, United States; ^2^Department of Pediatrics, University of California, San Francisco, CA, United States; ^3^Department of Medical Social Sciences and Institute for Innovations in Developmental Sciences, Northwestern University, Chicago, Il, United States; ^4^Department of Public Health Sciences, University of California, Davis, Davis, CA, United States; ^5^Community Environmental Health Program, College of Pharmacy, University of New Mexico Health Sciences Center, Albuquerque, NM, United States; ^6^Division of Child and Adolescent Psychiatry, Columbia University, New York, NY, United States; ^7^Department of Population Health, New York University, New York, NY, United States; ^8^Community Health Sciences, University of California, Berkeley, Berkeley, CA, United States; ^9^Department of Psychology, Penn State University, University Park, PA, United States; ^10^Prevention Science Institute, University of Oregon, Eugene, OR, United States; ^11^Center for Pediatric and Community Research, Avera Research Institute, Sioux Falls, SD, United States; ^12^Department of Pediatrics, University of North Carolina at Chapel Hill, Chapel Hill, NC, United States; ^13^Division of Research, Kaiser Permanente Northern California, Oakland, CA, United States; ^14^Department of Biostatistics and Epidemiology, RTI, Atlanta, GA, United States

**Keywords:** neurodevelopment, family, home, parenting, fetal programming

## Abstract

The family environment, with all its complexity and diverse components, plays a critical role in shaping neurodevelopmental outcomes in children. Herein we review several domains of the family environment (family socioeconomic status, family composition and home environment, parenting behaviors and interaction styles, parental mental health and functioning, and parental substance use) and discuss how these domains influence neurodevelopment, with particular emphasis on mental health outcomes. We also highlight a new initiative launched by the National Institutes of Health, the Environmental influences on Child Health Outcomes (ECHO) program. We discuss the role that ECHO will play in advancing our understanding of the impact of the family environment on children’s risk for psychiatric outcomes. Lastly, we conclude with important unanswered questions and controversies in this area of research, highlighting how ECHO will contribute to resolving these gaps in our understanding, clarifying relationships between the family environment and children’s mental health.

## Introduction

A child’s family environment, with all its complexity and diverse components, plays a critical role in shaping neurodevelopmental and psychiatric outcomes. The term “neurodevelopment” connotes the developmental unfolding of behavior, cognition, and emotion underpinned by brain maturation (1). Neurodevelopmental health reflects integrated brain–behavior patterns that promote flexible adaptation and regulation in response to shifting environmental demands. In contrast, neurodevelopmental disorders reflect delays or deviations in behavioral and psychological function due to atypicalities in brain development with associated impairment (2). Family environments with the varied opportunities, challenges, and experiences they provide, influence neurodevelopment with attendant effects on children’s motor and sensory development, temperament, cognitive abilities, and behavioral and emotional responses. Although the importance of the family environment in shaping children’s neurodevelopment is widely accepted, rarely has this been investigated systematically across the broad spectrum of family environmental domains or inclusive of a broad range of neurodevelopmental outcomes assessed simultaneously and prospectively. Richer examination of the environmental exposome is required to advance psychiatry (3). A newly launched multidisciplinary National Institutes of Health (NIH) initiative, the Environmental influences on Child Health Outcomes (ECHO) program, is well positioned to address these gaps and advance the knowledge base of child and adolescent psychiatry.

The NIH ECHO program is structured to study the influence of physical, chemical, biological, social, and behavioral exposures on child development within a large population of children in the United States (http://echochildren.org). It is a national, multisite study that brings together approximately 70 extant pregnancy and child cohorts and will comprise more than 50,000 children and their families (http://echochildren.org/pediatric-cohorts/). Building upon these successful extant cohorts with rich longitudinal databases and banked biospecimen repositories, ECHO promises to vastly expand our understanding of the determinants of neurodevelopment by creating large collective sample sizes, harmonizing data collection across studies, providing a shared measurement framework, and sampling across geographically, racially, ethnically, and socioeconomically diverse populations. Further, ECHO is leveraging the cohorts’ affiliated multidisciplinary research teams, with specific expertise in the study of children and families, to meaningfully advance knowledge of factors that promote optimal neurodevelopment and physical health of U.S. children. As such, the ECHO national consortium is designed to play a leading role in systematically examining how environmental, chemical, and social exposures from preconception to early childhood shape unfolding neurodevelopmental pathways and in understanding the etiology of comorbidities. Prior studies of the influence of the family environment have often focused on a single feature or have not robustly considered co-occurring exposures, such as toxicants (4). Thus, the robust nature of exposure measurement in ECHO provides an unparalleled opportunity to shed light on those features of the family environment that shape mental health outcomes, independently and in interaction with other environmental adversities. Further, ECHO has the capacity to incorporate genomics within its examination of family environmental risk factors for psychiatric disorders (5), in a manner that could advance population health through its focus on early neurodevelopment and opportunities to inform prevention efforts (6).

**Figure 1:**
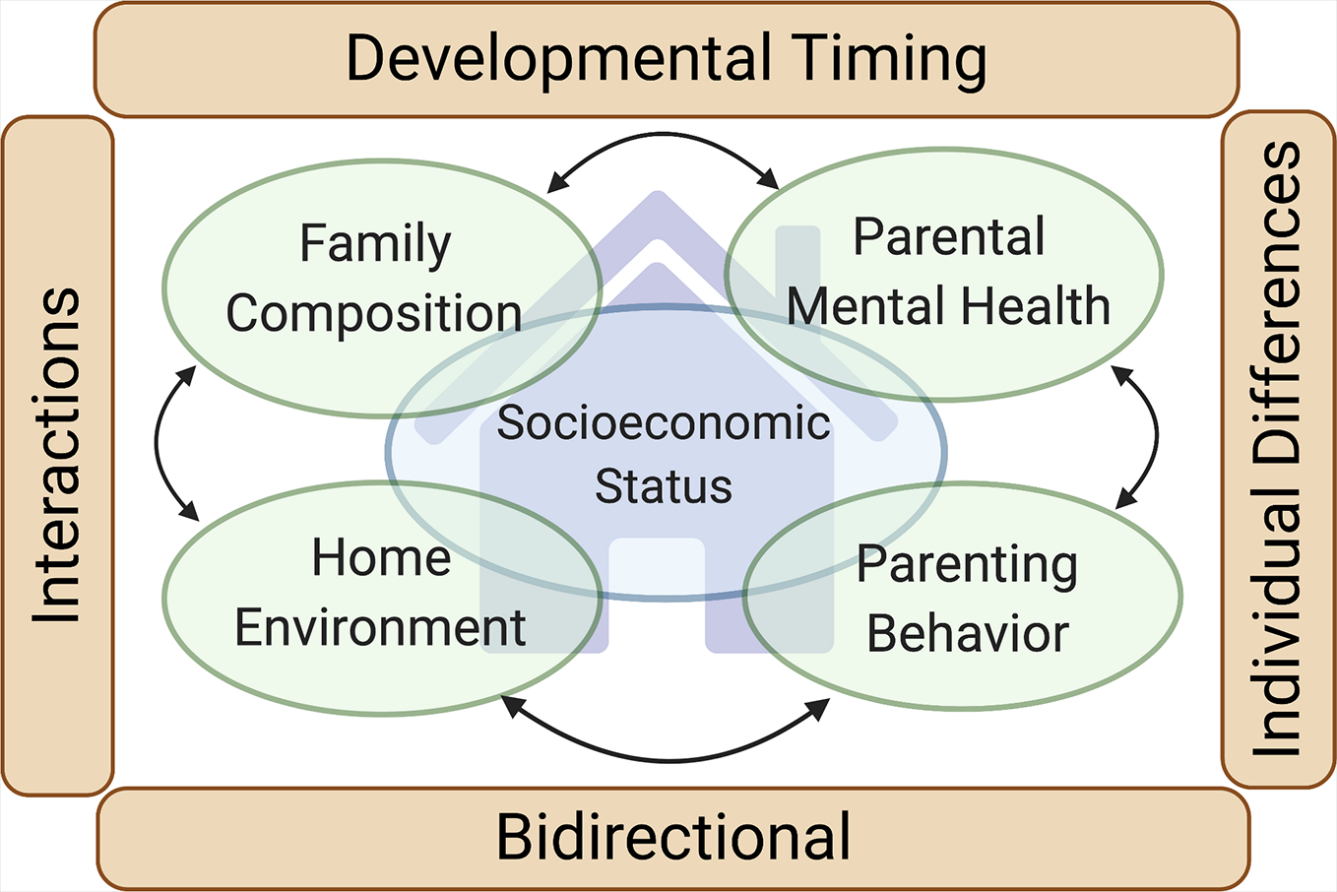
*Family environment and neurodevelopment*. Our review focuses on four primary domains of the family environment with a strong evidence base supporting their impact on the neurodevelopment of children (*green ovals*): Family composition, Home environment, Parenting behaviors and interaction styles, and Parental mental health and functioning (including parental substance use). Family socioeconomic status (*large blue oval*) intersects with each of these domains. Four cross-cutting themes impacting each of the four family environment domains are also highlighted (*brown rectangles)*: Developmental timing (the neurodevelopmental influence of the family environment varies depending on the developmental stage of the child), Individual differences (the neurodevelopmental influence of the family environment varies depending on traits of the child), Bidirectional (the family environment influences the child, but the child also influences the family environment), and Interactions (the neurodevelopmental influence of one family environment domain may be contingent on the others).

In this paper, we provide a brief review of the role that the family environment plays in shaping children’s neurodevelopment, with emphasis on factors contributing to risk for psychiatric disorders, acknowledging that the outcomes reviewed do not encompass all of neurodevelopment. Given the size and scope of this literature, we limit the scope of our review to focus on domains of the family environment with a strong evidence base supporting impact on offspring neurodevelopment, directing our review to the following primary domains of the family environment: family socioeconomic factors, family composition and home environment, parenting behaviors and interaction styles, parental mental health and functioning, and parental substance use. In considering associations between the family environment and neurodevelopment, we emphasize elements that the ECHO study is poised to address. We also highlight themes that cut across these family environment domains, considering, for example, bidirectionality of influences between the child and the family environment, as well as interactions across family environment domains (**Figure 1**). We begin with a review of the literature, covering established associations between the aforementioned domains of the family environment and child neurodevelopment and highlighting mechanisms underlying those associations, when known. We then present an overview of extant data from the ECHO cohorts with available data across those family environment domains. Lastly, we outline important unanswered questions and controversies in this research, highlighting how ECHO can contribute to resolving these critical gaps in our understanding of the role of the family environment and revealing opportunities for prevention of, and intervention for, neurodevelopmental psychiatric disorders.

## Review of the Influence of Family Environment Domains on Children’s Neurodevelopment

### Family Socioeconomic Factors

Roughly one in five children live in poverty in the U.S. (7), and in 2015, 51% of children in U.S. public schools were from low-income families (8). For decades, investigations focused on socioeconomic status (SES) in childhood indicate that children in lower SES families have poorer neurodevelopmental outcomes across multiple domains (9, 10), including global measures of cognitive performance like IQ and academic achievement (11), as well as specific cognitive domains such as language processing, working memory, and cognitive control (12, 13). Low SES is associated with externalizing disorders (10), including attention-deficit/hyperactivity disorder (ADHD) and disruptive behavior disorders (14), as well as internalizing behaviors such as anxiety and depression (15), though these latter associations have been less consistent (16). As with other mental and physical health outcomes, associations between SES and neurodevelopment are graded, with better functioning observed with increasing levels of SES (17), and the largest negative impact found in impoverished children (18).

Children born into low-SES families often encounter a broad range of adverse exposures that frequently coexist within a family or neighborhood of low SES. Early life adversities more common among low-SES children include material and nutritional deprivation (7), a less complex language environment, (8, 9) psychosocial stress (10), and more frequent exposure to toxic chemicals such as tobacco smoke and lead (17). Material deprivation refers to the impact of low SES on a parent or caregiver’s ability to provide a cognitively enriching environment in the form of toys and books, and also encompasses the quality and quantity of early educational opportunities (7, 9). Children from low-SES households are also more likely to experience psychosocial stressors, which include interpersonal violence, crowding, neighborhood violence and disorder, and disruptions in their relationships with caregivers (15). Additionally, low SES has been associated with reduced breastfeeding (19). Children from low-SES homes are thus less likely to receive breastfeeding’s beneficial effects on offspring IQ (20) and maternal well-being (21). Whether adversities that co-exist with low SES partially or fully mediate associations between a family’s SES and neurodevelopmental outcomes in the children is still unanswered. Studies such as ECHO with large and diverse sampling across developmental stages and exposures can examine mediation effects and determine the degree to which coexisting factors, including potential genetic liabilities, account for the influence of low SES on neurodevelopment.

Recently, investigators have identified neural correlates of lower SES in childhood, resulting in a nascent, brain-based understanding of how SES may “get under the skin.” While studies demonstrate the influence of lower SES on widely distributed brain regions (22), the most consistent effects are reported in brain areas implicated in executive function, language, and emotion processing (22). For example, several studies indicate that low SES is associated with alterations in the morphology of the prefrontal cortex (PFC), which broadly supports self-regulation, reasoning, and decision-making (23). Studies have also identified SES-related functional and volumetric alterations in the hippocampus and amygdala, regions critical to learning, memory, and threat/emotion processing. Structural brain differences in the frontal and temporal lobes have been shown to explain as much as 20% of the deficits in academic achievement among low-SES children (18). Though still a field in its infancy, neuroimaging studies of SES correlates may ultimately help inform interventions aimed at mitigating the adverse impact of low SES on neurodevelopment.

### Family Composition

Decades of research confirm that variations in family composition are associated with differential neurodevelopment. Children reared in two-parent families generally fare better than those raised in single-parent families in terms of cognitive, educational, and behavioral outcomes, and these associations are consistent across age groups (24). Much of this research has focused on children of divorce, who demonstrate poorer academic and emotional functioning compared to children in two-parent families (25, 26), though effects of family disruption may confound this observation. Moreover, transitions in and out of two-parent families are complex and appear to have unique and varying effects on child outcomes depending on the type of transition, the outcome of interest, and the population studied (see *Co-Occurrence and Context of Exposures* for further discussion) (27, 28). Increasingly, studies have considered the effects of parental separation due to incarceration on children’s emotional and behavioral health. A 2012 meta-analysis (29) concluded that parental incarceration was consistently associated with children’s antisocial behavior, but not with educational or mental health outcomes. This association persisted even when controlling for parental criminality and/or children’s antisocial behavior prior to parental incarceration. Additionally, birth order may be a significant factor in neurodevelopment. Firstborn children are often reported to have higher intelligence compared to their later born siblings (30, 31), but may also be at higher risk for certain negative outcomes, such as ADHD (32). Recent longitudinal research on birth order and cognitive outcomes, however, has yielded inconsistent or null findings (33).

A number of pathways have been proposed to explain how family composition affects children’s neurodevelopment. Associations appear to be largely mediated through other aspects of the family, many of which are covered in this review, including SES, parent–child interactions, and parental stress (25, 34, 35). For example, the importance of SES and parent–child interactions could be related to the availability of resources that may be less accessible to children in single parent and/or larger families. The same logic applies to birth order, such that parenting resources diminish with each birth, negatively affecting children later in the birth order (36) [although recent large studies have questioned this, due to finding no effect of birth order on IQ or personality (37)]. Whereas some research suggests that bivariate associations between family composition and child neurobehavioral outcomes are largely attenuated once individual, family, and neighborhood characteristics are accounted for (38), enduring, negative effects have nonetheless been shown for children of divorce across a range of outcomes (25, 39). Taken together, this body of research suggests that family composition plays a role in children’s neurodevelopment, though the effects appear to be connected to other factors such as culture, child sex, parental psychopathology, parenting behavior, and interparental conflict (40).

### Home Environment

Creating and maintaining an optimal, safe, supportive, and stimulating early home environment is considered an essential factor in promoting children’s well-being and long-term adaptive functioning, particularly in the presence of other environmental and contextual adversities (*e.g.*, poverty) (41). Characteristics of a child’s home environment and its quality can be classified into two broad categories: functional and structural. Functional characteristics specify the emotional climate and the quality of interactions and relationships among members of the household (*e.g.*, parent–child interactions and parental stress) and are covered elsewhere in this article. Here, we focus on structural characteristics, which reflect the physical, and often observable, aspects of the home environment (*e.g.*, residential crowding, quality of construction materials used in the house, proper facilities for food storage and hygiene, access to learning resources).

Low-quality structural characteristics of the home environment are often associated with adverse neurodevelopmental outcomes (42). As an example, many materials that have been or currently are used in building houses include heavy metals and other toxicants that can potentially jeopardize children’s safety by increasing their exposures, which negatively impact children’s cognitive and behavioral outcomes (43, 44). Some of these toxic chemicals include (1) polyvinyl chloride or other chlorinated plastics used in pipes, flooring and ceiling coatings, (2) volatile-organic and semi-volatile compounds in fabrics including carpets and furniture that can volatilize at room temperature and be inhaled and/or dermally absorbed, and (3) heavy metals (*e.g.*, arsenic, antimony, cadmium, cobalt, lead, mercury) found in a variety of forms such as paints, fabrics, thermostats, window blinds, and water pipes (45–47). Exposure to these toxic chemicals has been associated with neurodevelopmental outcomes such as ADHD and autism (46, 48–50).

Lack of infrastructure also remains a potential source of increased exposure for many populations in the US. For example, 14% of Native Americans lack access to regulated drinking water, while only 0.6% of the US population as a whole lacks that access (51). This increases risk for ingestion of pathogens or contaminants including heavy metals and industrial chemicals (similar to those linked above to diminished cognitive abilities, behavioral problems, ADHD, and autism) (51, 52). Lack of infrastructure can also result in homes with no or substandard heat and cooking sources associated with poor indoor air quality from particulate and other combustion product emissions. Combustion of dirty heating materials such as coal in substandard burners also increases the release of metals into the home environment (53, 54). Not only can exposures to toxicants described here contribute to adverse developmental outcomes, but the associated infrastructure deficits in the home environment may impact educational achievement if, for example, lack of electricity and heat inhibits the child’s ability to complete school assignments at home (55). Reasons for lack of infrastructure are varied and range from an inability to pay for service to a lack of services provided in low-density rural communities.

At even greater risk for exposure through substandard living environment and poor infrastructure is the staggering proportion of children in the United States who are growing up in families experiencing homelessness or housing insecurity. This number according to a 2014 report was estimated to be 2.5 million children, with 42% being under the age of 6, reflecting a large increase since 2006 (one in 50 children in 2006 compared to one in 30 in 2014) (56, 57). Homeless children with families fare better than those without other family members, yet both groups have poorer school performance than housed children; nevertheless, trajectories of school performance in homeless children can be heterogeneous (58). Children experiencing homelessness are also more likely to come from impoverished families with prolonged exposure to many factors associated with children’s behavioral and emotional problems such as domestic violence and abuse, parents’ drug abuse and/or poor mental health and extreme poverty (59). Children experiencing homelessness compared to housed children—even those living in poverty—are more likely to experience health problems (*e.g.*, asthma), developmental delays (*e.g.*, language and motor skill delays), hyperactivity and inattention, externalizing and internalizing behavioral problems, academic difficulties, and psychiatric disorders (*e.g.*, conduct disorder) (60–62). These problems may be further exacerbated by limited access to diagnostic and treatment facilities and the increasing stress on those systems as homelessness increases.

### Parenting Behaviors and Interaction Style

Parents’ interactions with their children are among the strongest proximal predictors of children’s neurodevelopment. One of the primary approaches to conceptualizing parenting has been a dimensional approach focused on continuous dimensions of specific parental behaviors, such as sensitivity, warmth, or control. A second approach has been to categorize parents into discrete parenting styles based on a combination of parenting dimensions, yielding parenting classifications such as authoritative, authoritarian, or permissive parenting (63, 64). In both approaches, a core parenting behavior in infancy and early toddlerhood is parental responsiveness, which has been defined as being accepting, nurturing, supportive, sensitive, and warm; conversely, low levels indicate insensitive, unresponsive, and rejecting parental behavior (64, 65). A recent meta-analysis of over 1,400 studies comprising more than one million children concluded that parental responsiveness has a significant positive association with the child’s later academic performance, as well as a protective effect against externalizing problems (including conditions like ADHD and conduct disorder), an association that was present for both maternal and paternal responsiveness (66). The important role of parental responsiveness has also been well-studied in the attachment literature with meta-analyses indicating a significant association between parental responsiveness and children’s attachment security (67).

In the decades following the seminal work of Bell (68, 69) that challenged the notion that children are merely passive recipients of parental socialization effects, there has been a growing body of research on the bidirectional nature of parenting behavior and children’s neurodevelopment. For example, parental responsiveness has been shown to lower risk for externalizing behaviors in the child, but this association was mediated, at least in one study, by the child’s performance on a battery of executive function tasks (70). In addition, children’s behavioral problems have been shown to have a reciprocal influence on negative parenting (71, 72). Such research on bidirectional associations has been strengthened by genetically informed studies that can measure, or control for, the role of shared genes between parent and child. For example, a longitudinal adoption study revealed a pathway from the biological mother’s ADHD symptoms to the adoptive mother’s low sensitivity that was mediated by the adopted child’s impulsive behavior (73). In other words, a child’s ADHD symptoms may give rise to ineffective parenting, rather than parenting giving rise to the child’s symptoms. A related but distinct construct is parent–child dyadic synchrony, which represents reciprocal adaptation of behaviors and affect between parent and child to form a single relational unit (74). A systematic review of children age 2 months to 5 years found that dyadic synchrony was associated with neurodevelopmental health, including higher IQ, attachment security, and fewer internalizing symptoms (75). Taken together, this work highlights the complexity of parent–child interactions and how parental behaviors and responsiveness may both influence, and be influenced by, the offspring’s behavior and neurodevelopment.

Studies that examine parenting after early toddlerhood and into adolescence often focus on the quality of parenting behavior, with a predominant focus on parental warmth, hostility or conflict, and control (76). Of these dimensions, parental hostility has been most commonly associated with adverse neurodevelopmental outcomes in children, including internalizing and externalizing behaviors and poorer executive functions (77, 78). Higher levels of parental warmth, on the other hand, have been linked with more positive neurodevelopmental outcomes like academic achievement, social competence, prosocial behaviors, emotional and behavioral regulation, and lower levels of externalizing behaviors (79). Interestingly, high levels of parental warmth and support have also been associated with higher levels of child inhibition across childhood (80, 81), suggesting that parental warmth may have different effects depending upon a child’s temperamental profile. The influence of parenting quality may also differ as a function of parent gender, with one study of Chinese children finding that father’s warmth, but not mother’s, was associated with child academic performance (82).

The parental behavior of “scaffolding,” defined as verbal or physical guidance from parents including support of autonomy and encouragement of problem solving and children’s choices (83, 84), has also been associated with higher levels of executive functioning during early childhood, including skills like working memory and cognitive flexibility (83), as well as better arithmetic skills (85) and improved early reading skills (86). The impact of scaffolding on neurodevelopment, however, may vary as a function of a child’s genetic background; one study found that maternal scaffolding was associated with higher levels of toddler behavioral problems for children at low familial risk, as indicated by the birth parents’ externalizing and internalizing problems, but with lower levels of behavioral problems for at high familial risk (87). Overall, parenting behaviors and interactional styles have robust associations with child neurodevelopment, but the complex interplay between parenting and children’s genetics require data from large-scale longitudinal studies such as ECHO.

### Parental Mental Health and Functioning

In recent decades, there has been a sharp increase in research examining the role of parental psychopathology, stress, and trauma during pregnancy and the effects on offspring neurodevelopment. This work, which has focused largely on maternal effects [with some exceptions noted (88)], is often termed “fetal programming” and draws on concepts from evolutionary biology (89, 90) to posit that maternal experience and resultant biochemistry provide crucial information to the developing fetus. Fetal programming may improve the fetus’ likelihood of survival but may also detrimentally affect other aspects of development and health. For example, a number of studies have suggested that prenatal maternal trauma (*e.g.*, war, divorce, physical and sexual abuse, natural disasters), particularly during the first trimester (91), contributes to neurobehavioral abnormalities in the child including motor impairments, emotional reactivity, hyperactivity, cognitive deficits, language delays, difficult temperament, social withdrawal, and aggression (91, 92), and may increase intrauterine inflammation with subsequent effects on offspring learning and memory (93). These neurobehavioral effects may persist into adulthood, with some studies linking prenatal trauma to an increased risk of post-traumatic stress disorder, schizophrenia, depression, and/or substance abuse disorder in the offspring (94, 95). Prenatal maternal stress need not be “traumatic” to impact the offspring. Accumulating research points to effects of a variety of indices of maternal stress (*e.g.*, self-report of stressful life events or perceived daily stress) on offspring neurodevelopmental outcomes (96, 97), including increased infant reactivity, poorer emotional and behavioral regulation, and differences in brain structure and function in infancy and childhood (96, 98). It is important to note, however, that mild to moderate exposure to stress during pregnancy may not always be detrimental to the fetus. Indeed, some studies suggest it is associated with better offspring mental and psychomotor development (99), particularly when exposure occurs later in pregnancy (99) or when there are congruent levels of adversity during pregnancy and postpartum (96).

In contrast to fetal programming, genetic transmissions are also critical to consider. Indeed, associations between prenatal maternal stress and offspring neurodevelopment may in part, or even fully, be accounted for by shared genetic inheritance (100). Testing these competing hypotheses requires longitudinal assessments, coupled with detailed phenotypic, genetic, and epigenetic assays—a breadth and depth of data that, prior to ECHO, has rarely been available, particularly in U.S. cohorts.

In addition to prenatal maternal stress, maternal psychopathology, including depression, has been repeatedly associated with negative effects on offspring’s physical, cognitive, and affective development. Perinatal maternal depression (PMD) affects 11.9% of women worldwide, with prevalence rates ranging from 9 to 22% for prenatal depression and 7 to 19% for postnatal depression (101, 102). PMD increases risk for pregnancy complications, preterm birth, intrauterine growth restriction, and low birth weight (103, 104), all of which may impact the child’s subsequent neurodevelopment. Indeed, infants of prenatally depressed mothers show increased negative affect, irritability, growth retardation, and delays in cognitive, motor, and emotional development (105), as well as altered psychophysiological measures (*e.g.*, vagal tone) and early brain development (*e.g.*, increased right frontal electroencephalogram (EEG) activity and altered amygdala microstructure and functional connectivity) (106–108). Though only a handful of longitudinal studies have been conducted, they suggest that PMD exposure increases risk for externalizing problems in childhood and for depression in adolescents, potentiating risk for long-term psychiatric consequences (109–111).

During the postnatal period, parental stress and psychopathology also adversely affect offspring neurodevelopment, increasing risk for anxiety, emotional reactivity, cognitive deficits, language delays, aggression, substance abuse, and a host of other mental health problems throughout life (105, 107). Adverse neurodevelopmental effects to the offspring have been implicated from a broad range of parental psychopathologies (*e.g.*, depression, bipolar disorder, and psychotic disorders), as well as non-psychiatric illnesses (*e.g.* cancer, chronic inflammatory disorders) (112), as demonstrated in large-scale, national registries (113, 114). Although pre- and post-natal exposure to maternal stress and psychopathology both impact offspring neurodevelopment, mechanistically they differ. Prenatal maternal stress is thought to exert its intergenerational influence primarily through its effects on the placenta, hypothalamic^–^pituitary^–^adrenal (HPA) axis, and inflammation (115, 116). For example, when the HPA axis is activated in distressed mothers, peptides such as corticotropin-releasing hormone are released by the placenta, thereby exacerbating the effects of stress on both the mother and fetus (117, 118). Similarly, maternal cytokines increase with maternal stress, and some may pass through the placenta, thereby creating an inflammatory environment for the fetus (119). Conversely, the mechanisms by which postnatal maternal stress and psychopathology influence offspring neurodevelopment are more behavioral in nature, with adverse effects on caretaking (*e.g.*, maltreatment, malnourishment, physical and sexual abuse) and mother-infant attachment. However, intriguing experimental research also suggests that maternal stress physiology is transmittable to her child’s physiology through touch, even when the baby cannot see the mother’s face, suggesting that maternal-to-infant transmission of stress may involve mechanisms beyond overt parent–child interactions (120, 121). These mechanistic pathways, however, are largely theorized and do not yet have a strong evidence base. Parallel to evidence from the parenting literature, there is also evidence for transactional or bidirectional relations between parental and child mood and behavior, beginning in early childhood, adding complexity to the process of understanding postnatal parental mental health impacts on children (122, 123). Large-scale longitudinal studies such as ECHO that measure maternal mental health during pregnancy and parental mental health postnatally will help disentangle the effects of parental mental health on child neurodevelopment.

### Parental Substance Use

There is a vast literature on prenatal exposure to both licit and illicit drugs and adverse neurodevelopmental outcomes, including alcohol, smoking, cocaine, methamphetamine, opioids, and marijuana. The common and most robustly demonstrated effect across substance exposure type is an association with offspring behavioral disinhibition and associated clinical patterns (124, 125). Behavioral disinhibition encompasses trait-like deficits in cognitive and behavioral control that are evident from the first years of life (126, 127). Behavioral disinhibition includes impairments in top-down cognitive control processes subserved by the lateral prefrontal and anterior cingulate cortices, impaired development of bottom-up arousal and reward centers in the nucleus accumbens and orbitofrontal cortex, as well as memory-related hippocampal structures (128). It presages a range of clinical problems including externalizing behaviors and related disorders and poor health and social functioning, more generally *via* its risk-taking and impulsive features (129, 130). Prenatal alcohol and cigarette exposure have the most strongly demonstrated and the most coherent patterns in relation to the development of disinhibition beginning in early life. Those links and their association with offspring substance use and severe antisocial behavior are particularly powerful as they highlight an intergenerational mechanism of transmission (131, 132). We highlight some of these links illustratively below.

Prenatal alcohol exposure (PAE) is known to impact a range of neurodevelopmental domains such as attention, behavior, academic achievement, cognition, language development, memory, and motor and visuospatial development in children. The effects of PAE on neurodevelopment appear to vary by the dose, pattern, and timing of exposure during gestation, and the magnitude of the effect may vary by neurodevelopmental domain. On one end of the spectrum, heavy PAE is the cause of Fetal Alcohol Syndrome (133), which is characterized by neurological abnormalities (*e.g.* seizures), developmental delays, intellectual deficits, growth deficits, and distinct facial features. Heavy PAE has been linked to deficits in children’s performance, verbal IQ, and interpersonal skills (134, 135) and there is robust evidence of its links to behavioral disinhibition (*e.g.*, hyperactivity, attentional deficits, and delinquency) (135). The findings for lower levels of PAE have been much more inconsistent—both deficits (136, 137) and benefits (138) in general cognitive functioning have been noted, and several studies have found no relationship with child behavioral problems (139). These inconsistencies may be due to a lack of adequate control for potential confounders such as home environment or SES.

Alcohol’s toxic effects on fetal neurodevelopment can occur through direct or indirect pathways. Directly, alcohol crosses the placenta and can impair neuronal maturation. Alcohol may also inhibit the transfer across the placenta of essential cofactors and/or antioxidants necessary for neuronal maturation (140). Alcohol induces oxidative stress through the generation of free radicals, hypoxia, and altered metabolism—all of which can lead to significant oxidative damage in fetal tissue and reductions in oxidant defense mechanisms in the fetus (141). Alcohol-induced oxidative stress and oxidative tissue damage have been linked to developmental delays (142). As alcohol is metabolized, free radicals are produced, which target polyunsaturated fatty acid chains in brain tissue and membranes, damaging developing neurons and resulting in neurodevelopmental impairment (143) or, in more severe cases, inducing uncontrolled apoptosis and fetal brain damage (144–146).

Prenatal exposure to opioids, in the majority of children, gives rise to neonatal abstinence syndrome (147), characterized by hyperactivity of the central and autonomic nervous systems. While these abstinence effects are short-lived, epidemiologic research on the longer-term effects of *in utero* opioid exposure suggests a number of cognitive, motor, and behavioral deficits such as inattention and an increase in ADHD (148). Animal models further suggest a significant impact on fetal development including disruptions of neuronal migration and cell survival (149), decreases in dendrite length and branch number in pyramidal neurons in the somatosensory cortex (150), and increased neuronal apoptosis resulting in memory deficits (149).

In multiple independent studies using a variety of study designs, prenatal tobacco exposure (PTE) has been linked to behavioral disinhibition patterns beginning in early childhood (*e.g.*, atypical externalizing trajectories, heightened negative affectivity), across childhood and adolescence (*e.g.*, oppositional defiant disorder and conduct disorder) and into adulthood (*e.g.* criminal behavior, substance abuse) (125, 151, 152). Associations of PTE with disruptive behavior and impaired executive function under motivational conditions are consistently found as well as comorbid disruptive and ADHD syndromes, with weaker or inconsistent links to ADHD alone and “cool” executive functions (153–155). The biologic plausibility of these patterns is undergirded by evidence from basic neuroscience (156), and from mechanistic studies demonstrating exposure-related alterations in neural organization (*i.e.*, atypical white matter developmental trajectories in infants) and structural and functional exposure-related patterns in adolescence that have been implicated in antisocial behavior and substance abuse (*e.g.*, orbitofrontal cortical thinning and functional decrements in response in the ventral striatum during reward anticipation).

As with PAE and PTE, studies examining prenatal cocaine, methamphetamine, opioid, marijuana, and others (*e.g.*, MDMA), demonstrate widespread effects on neurodevelopment from infancy through later childhood. Effects have been observed on measures of attention, working memory and executive function, speed of processing, verbal ability, fine motor ability, and semantic and episodic memory (157). Longer-term effects have been observed in a number of studies, with a recent systematic review of prenatal cocaine exposure indicating small-to-moderate effects on language and memory measures into adolescence and young adulthood (158). Additionally, effects on children’s brain structure and function have been observed with some specificity. For example, methamphetamine exposure is associated with reduced volume and/or surface area of the basal ganglia and hippocampus (159), and cocaine exposure with reduced volume in the cerebellum, corpus callosum, and occipital and parietal lobes (160). Systematic review of the small neuroimaging literature of prenatal drug exposure tends to indicate vulnerability in dopaminergic projections within the mesocortical system as a potential common pathway of effects (160).

Links between prenatal substance exposure and behavioral disinhibition patterns and their clinical/functional correlates (*e.g.*, antisocial personality disorder, criminality) are robust and independent of obvious confounders, including SES, parental mental health and substance abuse history, and quality of the family environment (161). However, prenatal substance use may have underlying genetic substrates predisposing to behavioral disinhibition (*i.e.*, impulsive, nonconforming, sensation-seeking, and risky behavior), which would make these patterns attributable to genetic transmission rather than teratologic effects (162–164). As a result, statistical control alone is inadequate for separating direct casual effects from family variation (165, 166). This issue has been addressed most rigorously in relation to prenatal smoking and behavioral disinhibition patterns, where the Surgeon General has assessed evidence as “suggestive but not conclusive” (167). Studies have creatively used quasi-experimental, genetically-sensitive designs to tease apart familial and teratologic mechanisms. Most influential have been large population-based studies using discordant sibling designs leveraging within family differences in sibling exposure to test whether individual level exposure has an independent effect (168). The majority of these studies have failed to show independent exposure effects, leading to conclusions that exposure is merely a marker for genetic risk (168–170). The conclusion that these associations are spurious, however, is called into question by evidence that independent exposure effects are present when behavioral genetic designs are coupled with high quality exposure measurement (165).

In addition to maternal prenatal substance use, contributions of postnatal substance use to offspring neurodevelopment have been examined, particularly as a means of comparing putative teratologic vs. family relationship correlates (171). Although early studies took an either/or approach, it is increasingly evident that the neurodevelopmental impact of exposure timing must be understood in a more nuanced manner. Both additive and interactive effects have been demonstrated, likely reflecting a combination of teratologic effects, disruptions in home environment and parental responsiveness, and heritability (125, 172). ECHO’s large sample size, robust pre- and postnatal exposure measurement and varied participant cohort designs provide a rare opportunity to disentangle these pathways.

## Co-Occurrence and Context of Exposures

Adverse exposures within the family environment are often situated within a landscape intermixed with additional exposures (3). For example, hazardous waste sites, chemical plants, smelters, and other polluting industries are disproportionately situated near low-SES communities (173). It is thus possible that the influence of low SES on neurodevelopment may, in some cases, be mediated or exacerbated by chemical toxicants more common in a low SES environment. This example of “double jeopardy” arises not only from the intersection of exposures across domains (SES and chemical toxicants), but also in how exposures can synergistically increase health risks. For example, maternal periconceptional nutrition is influenced by family SES and may protect high SES children from the adverse impact of pesticides on risk for autistic spectrum disorders (ASD) and intellectual development (174). Therefore, low-SES children may not only be more likely to be exposed to some chemical toxicants, but their neuropsychiatric sequelae may be exacerbated by other risk factors disproportionately represented in a low SES family environment. Rich exposure measurement in ECHO will advance our ability to conduct well-powered examinations that control for confounding by other exposures and/or allow for the determination of cumulative and interactive effects, although careful attention will need to be paid to modeling and interpretation given the complexities of multi-exposure models (175). In addition, the public health significance of a given exposure may vary substantially by neurodevelopmental outcome. Therefore, in addition to considering multiple exposures, ECHO presents an opportunity to also examine multiple-outcomes, and pinpoint the specificity of cumulative and interactive effects of family context exposures (175).

It is also important to note that associations between neurodevelopment and the family environment may be contingent upon the culture in which the family is situated. For example, overcrowding (see *Home Environment*) has been shown to predict negative neurodevelopmental outcomes for children in Western cultures (176), which are characterized by individualism and a high demand for personal space and privacy; however, a high number of people living in a household may benefit children’s socio-emotional development in cultures that emphasize collective child-rearing and have a higher tolerance for crowding. Similarly, associations between neurodevelopment and the home environment may vary in urban vs. rural settings (177). Children raised in more remote areas, where they have broad access to natural settings for play and socialization, may be less affected by limited access to “standard” learning materials—access to natural settings that increase free play and peer interactions may even promote a child’s socio-emotional and cognitive competence (178).

Another example of the importance of culture and context in shaping neurodevelopment is evident in the influence of family composition and parental transitions. As noted above, children reared in two- *vs.* single-parent families generally fare better on neurodevelopmental outcomes (see *Family Composition*). However, recent research using birth cohort data from the Fragile Families and Child Wellbeing Study revealed that among Black children and girls (compared to other racial/ethnic groups and boys) transitions from two- to one-parent families negatively influenced cognitive achievement, but among White children (regardless of gender) the negative impact was more evident with behavioral outcomes (28). Conversely, transitions *into* two-parent families had adverse behavioral effects on Latino children. Findings from the Boricua Youth Study have similarly reinforced the importance of context in moderating the relationship between family composition and neurodevelopment, demonstrating, for example, that transitions from two- to single-parent families have a greater impact on children’s internalizing symptoms among Puerto Rican children living in Puerto Rico relative to those living in the New York (179). Rich representation in ECHO, and sufficient power should advance understanding in these realms.

In addition, disparities by sex and gender in rates of various neurodevelopmental disorders, as well as discoveries of sex-specific biological mechanisms of exposure transmission and gender as a social determinant of health and moderator of sex effects, point to the important role of sex and gender in the context of the associations described above (180). However, sex-specific findings across studies are remarkably mixed and contractictory (181), and fathers’ roles are sorely understudied, limiting progress in this domain. A sex- and gender-informed perspective is required for advancements in discovery, enhanced relevance of scientific findings, and improved patient care (181). Large samples of boys and girls, assessing both maternal and paternal contributions to children’s environment and child sex- and gender-specific vulnerabilities or resiliencies to exposures, are needed.

## Developmental Timing and the Family Environment

Although the family environment plays an important role in neurodevelopment across the life course, there is longstanding acknowledgement of the importance of critical and/or sensitive periods of development (182, 183), during which brain and physiologic systems are particularly plastic. This plasticity to environment is largely adaptive, although in contexts with significant adverse exposures it can contribute to aberrant neurodevelopment (184). The embryonic and fetal period may be particularly important sensitive windows during which the effects of adversity can be transmitted across generations, and prenatal programming may begin to set the stage for subsequent psychopathology. Postnatally, early childhood (185) and the pubertal period (186, 187), when substantial neurobiological and hormonal changes occur rapidly, may also be key periods of sensitivity when the effects of SES, parenting, and the home and external social environment have substantial impacts across a range of neurodevelopmental outcomes. Outside of animal models, developmental research to date has rarely had the capacity to systematically test for sensitive periods because studies typically focus on a particular developmental period, pre–postnatal exposures are highly correlated (*e.g.* harsh parenting or parental depression may not be limited to a specific developmental period), and/or sample sizes are inadequate for examining within group differences in exposure timing. In light of the interactions between family/social and chemical stressors described earlier, and the accumulating evidence for sensitive periods for toxin effects on neurodevelopment (188, 189), attention to developmental period is imperative. ECHO will be uniquely positioned to examine these questions with its large sample size, common protocol and extensive high-quality data on a range of exposures from preconception through pregnancy, early-to-mid childhood, and through adolescence. In doing so, ECHO will deliver more mechanistic and developmentally-informed insights into contextual factors—including the family environment—that influence child neuromaturation and identify modifiable elements of the environment that can promote optimal health and development with an eye towards targeted prevention as early as possible in the clinical sequence.

## Echo and the Family Environment

The ECHO study is poised to address some of the major gaps in the examination of the complex influences of the family environment on psychiatric outcomes, beginning in utero. In developing the ECHO study, the National Institutes of Health (NIH) solicited applications from existing cohorts to address the influence of pre, peri, and postnatal environmental exposures on childhood development. The ECHO study can thus leverage *existing* data (*i.e.*, data collected by ECHO cohorts prior to the onset of the ECHO study, or pre-ECHO-wide data), as well as *prospective* data (*i.e.*, data collected by ECHO cohorts after the onset of the ECHO study, or the ECHO-wide protocol) to lead inquiry into pediatric health and disease. The consortium prioritizes five primary outcome domains: neurodevelopment (highlighted here), as well as pre-/perinatal outcomes, airway, obesity, and positive health. The consortium sample of children has roughly equal numbers of male and female children, and will collect data on gender identity across development, which will advance consideration of sex and gender in the etiology and prevalence of those outcomes.

Existing data that ECHO cohorts collected prior to the onset of ECHO (pre-ECHO-wide data) followed protocols specific to each cohort’s scientific aims. Conversely, *prospective* ECHO data (ECHO-wide protocol) will include a systematic protocol with uniform data elements across all ECHO sites, harmonizing data collection and facilitating shared, or pooled, data analysis. *Prospective* ECHO data collection is ongoing and will continue through 2023. A description of the ECHO-wide protocol is detailed here: https://echochildren.org/echo-program-protocol/

**Figure 2:**
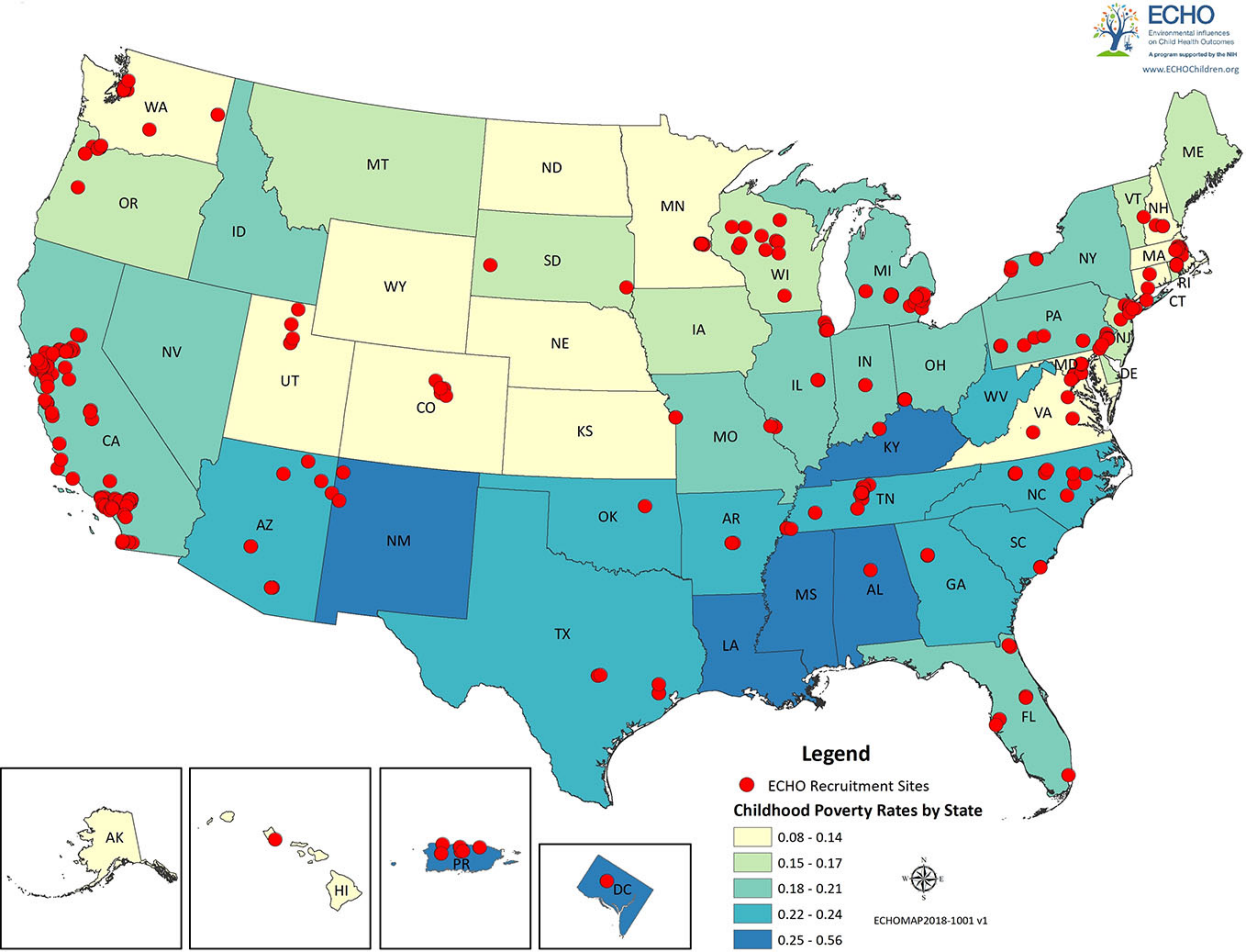
*ECHO recruitment sites*. Red dots represent the locations of the recruitment sites for the ECHO cohorts. States and regions are color-coded by poverty rates.

ECHO intends to make both the existing and prospective data (*i.e.* pre-ECHO-wide data and ECHO-wide protocol) available to the consortium of investigators within ECHO, as well as to the broader scientific community in order to maximize impact, intellectual engagement, and data utilization. Herein, we present an overview of existing ECHO data (pre-ECHO-wide data) within the family environment domains that we have reviewed (**Tables 1A–C**), additional family environment variables not directly discussed in this review (**Supplementary Table S1**), as well as the ECHO recruitment sites and geographic diversity of its cohorts (**Figure 2**).

**Table 1A:** (A–C) ECHO cohorts and sample size with family environment survey data.

Survey	Number of cohorts	Preconception	Prenatal	Infancy	Early Childhood	Middle Childhood	Adolescent
***Socioeconomic Status***							
*Education*	69	16	48	27	35	18	5
*Employment status*	55	16	44	28	34	15	5
*Occupation*	47	15	25	26	33	17	3
*Household income*	59	12	41	36	40	23	6
*Economic stress*	30	6	25	22	20	10	5
***Family Composition***							
*Social networks*	18	3	13	9	10	4	3
*Marital status*	61	13	44	26	36	19	5
***Parental Experiences***							
*Adverse childhood events*	16	NS	NS	NS	NS	NS	NS
*Maternity leave*	10	NS	10	NS	NS	NS	NS
*Interpersonal violence*	23	4	19	14	15	7	4
***Parental mental health*** ***and functioning***							
*Psychological stress*	52	13	32	31	32	18	5
*Family psychiatric history*	27	4	9	13	18	11	2
*Racial discrimination*	12	4	9	3	4	3	2
***Parental substance*** ***use***							
*Alcohol use*	58	24	55	18	23	12	2
*Tobacco use*	61	24	58	27	30	16	7
*Prescription drug misuse/abuse*	32	17	28	15	20	10	2
*Illicit drug use*	45	11	41	16	20	10	2
***Table 1B***							
***Socioeconomic Status***							
*Education*	50	8	25	12	22	12	3
*Employment status*	32	4	19	10	17	8	2
*Occupation*	32	8	11	11	20	11	1
*Household income*	35	9	16	17	24	15	3
*Economic stress*	8	3	2	6	7	4	0
***Family Composition***							
*Social networks*	2	1	0	1	1	1	0
*Marital status*	30	2	19	9	14	6	1
*Interpersonal violence*	5	1	0	4	4	4	0
***Parental Experiences***							
*Adverse childhood events*	1	NS	NS	NS	NS	NS	NS
*Racial discrimination*	2	1	0	0	0	1	0
***Parental mental health*** ***and functioning***							
*Psychological stress*	10	2	3	6	8	5	0
*Family psychiatric history*	23	3	4	13	18	9	0
***Parental substance*** ***use***							
*Alcohol use*	18	11	12	5	12	4	1
*Tobacco use*	27	11	18	9	17	9	4
*Prescription drug misuse/abuse*	16	3	4	4	12	4	1
*Illicit drug use*	17	4	5	4	12	4	1
***Table 1C***							
***Family Composition***							
*Number and age of siblings*	46	N/A	NS	36	35	20	5
*Number of children under 18 in household*	49	N/A	NS	38	40	22	7
*Number of adults living in primary* *residential household*	46	N/A	NS	34	37	20	6
*Number of siblings*	46	N/A	NS	36	35	20	5
*Birth order*	44	N/A	NS	NS	NS	NS	NS
***Home Environment***							
*Neighborhood violence/crime*	12	N/A	NS	11	12	7	4
*Urbanicity*	12	N/A	NS	12	12	10	6
*geocoded address*	29	N/A	NS	27	24	16	4
*Street Address*	64	N/A	NS	63	55	29	8
***Caregiving Context***							
*Reports of parenting or* *parent–child interactions*	23	N/A	NS	18	18	14	4
*Observational coding of* *parent–child interactions*	21	N/A	NS	17	16	10	2
*Sibling relationships*	8	N/A	NS	8	8	6	0
*Family dynamics*	14	N/A	2	11	11	12	2
*Childcare arrangement/daycare attendance*	54	N/A	NS	45	46	17	5

The number of ECHO cohorts with existing survey data from at least one time point from **(A)** Mother reporting on herself; **(B)** Father, or mother, reporting on the father; and **(C)** Father, or mother, reporting on the child.

The number of cohorts collecting this information at various life stages of the child was also enumerated.

N/A not applicable, primarily information about children in preconception phase. NS, Not surveyed of the ECHO cohort.

We compiled pre-ECHO-wide data data from ECHO cohorts from the ECHO Data Analysis Center (ECHO-DAC), which conducted a survey *via* secure, web-based Voxco (http://www.voxco.com/) of the specific survey instruments used with mothers, fathers, and children at each life stage by each ECHO cohort. The survey was sent to all ECHO cohort principal investigators, all of whom completed it. Information from this survey was tabulated by the ECHO-Data Analysis Center (ECHO-DAC). Based on these data sources, **Tables 1A–C** provide the number of ECHO cohorts with data available in each of the family environment domains. These estimates assume that there is sufficient similarity across instruments to allow harmonization of data across ECHO cohorts. The ECHO surveys were conducted between January and March 2018, and the surveys can be assessed through the ECHO metadata catalog (http://www.echoportal.org/); registration is required.

Out of 70 ECHO cohorts, the vast majority (between 55 and 69 cohorts) included primary measures of SES (income, education, occupation and employment status) and family composition (marital status, 61 cohorts) (**Table 1A**). A large number of cohorts also include measures of maternal psychosocial stress (52 cohorts) as well as alcohol and tobacco use (58 and 61 cohorts, respectively). While a smaller number of cohorts included measures of maternal social networks, maternal adverse childhood events, and exposure to interpersonal violence, these exposures may nonetheless be sufficiently well powered in an ECHO-wide analysis. Many ECHO cohorts also include information on father’s SES and marital status, with a smaller number including measures of paternal psychosocial stress, family psychiatric history, and substance use (**Table 1B**). Few cohorts included measures of father’s social networks, exposure to interpersonal violence, adverse childhood experiences, or racial discrimination. Finally, there is broad representation across the ECHO cohorts of additional measures that further contextualize the child’s environment including: detailed information on family composition such as household size and birth order, as well as parent–child relationship measures and childcare arrangements (**Table 1C**).

**Figure 2** displays the location of the recruitment sites for the ECHO cohorts and was generated in ARCGIS version 10.3 (https://www.arcgis.com/features/index.html). Information on the location of the ECHO cohorts and their recruitment sites was assessed by a Voxco survey sent by the ECHO-DAC to all cohort principal investigators; all cohorts completed the survey. States are color coded by childhood poverty rates, which were obtained on 2/14/2018 from the US Census Bureau (https://www.census.gov/topics/income-poverty/poverty.html).

To illustrate the utility and strength that ECHO will provide for the field, we highlight an ongoing ECHO analysis examining the complexities inherent to the search for causes of autism. Meta-analysis indicates a null association between maternal cigarette smoking and increased risk for autism spectrum disorder (ASD) (190). These studies, however, have been limited, not adequately controlling for confounders or inappropriately controlling for intermedia. At the same time, over a dozen studies have reported significant associations between prenatal exposures to air pollutants and increased risk for ASD (191). Because the components of air pollution and cigarette smoke share thousands of chemicals in common, this discrepancy raises a conundrum—is it credible that air pollution, which represents a generally lower level of exposure, has a causal effect on ASD while cigarette smoking, which generally includes much higher concentrations, has no impact?

Far better than any single or small set of studies, ECHO can effectively address this issue. Indeed, an ECHO effort is currently underway to conduct a multi-site examination of the association between prenatal cigarette smoke and the risk for ASD that will systematically account for a wide range of potential confounders within the family environment, including sociodemographic factors, health care access, maternal health conditions and her use of medications and other substances, family history of developmental and psychiatric conditions, and other environmental pollutants such as pesticides and particulate matter. This coordinated, distributed analysis will culminate in a statistically powerful meta-analysis of harmonized data from across the country, which has previously been difficult to achieve. Future ECHO analyses in this arena will integrate genomics to further advance this inquiry.

## Conclusions

Despite the significant advances made in understanding the relationship between family environment and neurodevelopment, important gaps persist that ECHO is well positioned to address. First, prior research has relied heavily upon cross-sectional approaches and/or retrospective reporting on the family environment, many of which were not designed *a priori* to examine the impact of the family environment on neurodevelopment. Prospective, longitudinal methodologies, and high-quality measurements, such as have been and will be used in ECHO, will allow for in-depth examination of specific factors influencing offspring neurodevelopmental trajectories with more precise estimates of how these factors mediate changes in ongoing developmental processes and across multiple potential sensitive periods of development. Second, many studies of the family environment have relied on convenient or highly-selected samples, calling into question their generalizability to the broader US population. ECHO, with its large, diverse cohorts from across the US, will address generalizability and will also foster more rigorous methods to strengthen causal inferences, such as propensity score analysis and/or repeated measures analyses (*e.g.* using within-child, fixed effects modeling to partially account for endogeneity).

With increasing evidence that the origins of mental health, disorder, and corollary neurodevelopmental disruptions begin even before birth (192), the large-scale, prospective consortium approach of ECHO is vital to explicating the contribution of a broad range of perinatal and early childhood environmental exposures to these pathways (79). Exposures are multifaceted and complex and their effects may vary depending on when they occur during development. Within the ECHO framework, we have a roadmap for deepening and broadening characterization of the role of multiple features of the family environment in the developmental unfolding of mental disorders. Systematic specification of malleable family processes that underlie emergent and sustained mental health risk is the crucial next step towards early life prevention of developmental psychopathology and its comorbidities.

## Author Contributions

Each of the authors contributed to conceptualizing, drafting, and editing the manuscript.

## Funding

Research reported in this publication was supported by the Environmental influences on Child Health Outcomes (ECHO) program, Office of The Director, National Institutes of Health, under Award Number U2COD023375 (Coordinating Center), U24OD023382 (Data Analysis Center), and UG3OD023271, UG3OD023365, UG3OD023344, UG3OD023328, UG3OD023332, UG3OD023389, UG3OD023279, UG3OD023348, NIEHS (National Institute of Environmental Health Sciences R01-ES015359; P30-ES023513, UG3OD023305 (NYU), U24OD023319.

## Disclaimer

The content is solely the responsibility of the authors and does not necessarily represent the official views of the National Institutes of Health.

## Conflict of Interest

JP has received research support from Takeda (formerly Shire) and Aevi Genomics and consultancy fees from Innovative Science.

The remaining authors declare that the research was conducted in the absence of any commercial or financial relationships that could be construed as a potential conflict of interest.
