# Supplementary material for: Family Environment, Neurodevelopmental Risk, and the Environmental Influences on Child Health Outcomes (ECHO) Initiative: Looking Back and Moving Forward
Source: Front Psychiatry. 2020 Jun 19;11:547. doi: 10.3389/fpsyt.2020.00547 (PMC7318113; doi:10.3389/fpsyt.2020.00547)
Supplement: Supplementary file 1 [file DataSheet_1.pdf]

Supplementary Table S1: Available ECHO cohorts and samples with Family and Neurodevelopment survey data. The table covers variables not discussed in the paper, but for which information has been collected by ECHO cohorts. The number of samples that collected a survey instrument at least one time point from the father or mother about the father. The number of cohorts collecting this information at various life stages of the child are also enumerated

Survey	*Number of studies*	*Pre-conception*	*Prenatal*	*Infancy*	*Early Childhood*	*Middle Childhood*	*Adolescent*
Maternal Neighborhood violence	21	5	17	16	15	8	4
Paternal Health insurance coverage	14	2	10	3	11	2	1
Paternal Neighborhood violence	5	2	1	4	4	4	0
Child Health insurance coverage	39	N/A	NS	25	31	13	5
Child Social networks (friends)	15	N/A	NS	10	13	12	6
Child Kangaroo care	6	N/A	NS	NS	NS	NS	NS
Child Parent-child feeding interactions	18	N/A	NS	18	NS	NS	NS
Child Diet	44	N/A	NS	39	34	21	4
Child Breastfeeding	63	N/A	NS	60	32	6	0
Child Formula feeding	58	N/A	NS	56	27	6	0
Child Age at introduction of solid foods	38	N/A	NS	36	22	6	0
